
JOURNAL INFORMATION
==============================
NLM Title Abbreviation: J Comp Med Vet Arch
NLM Journal ID: 17840220R

The Journal of Comparative Medicine and Veterinary Archives

Publisher: Biomedical Journal Digitization Project

ARTICLE INFORMATION
==============================
PMCID: PMC9342038
PMID: 36393173
Article version: 1
Subjects: New Inventions

New Inventions

Print publication date: 1896 Aug
Volume: 17
Issue: 8
First page: 648
Last page: 648
License: This work is free of known copyright restrictions. For more information, please see PMC Back Issue Digitization.
License URL: https://creativecommons.org/publicdomain/mark/1.0/

==============================

### NEW INVENTIONS.

A veterinary obstetrical instrument, consisting of a handle-shank, with a guide-loop at one end, at the other a circular group of divergent, normally separated spring-wire jaws, having terminal loop-eyes, a draw-cord passing through the eyes, a spider adjusting-head arranged to move entirely within the group of wire jaws, and so arranged to collapse the spring-wire jaws within a prescribed compass, and to hold the same rigid during insertion.

A device for ringing hogs.

A horseshoe, with flanges at the toe and heels, made wider at one end than the other, so dovetailed as to lock with a key device, so as to be removable when desired.

A horseshoe with a detachable heel-bar.

An automatic stock watering-trough.

A knock-down feed-box.

A veterinary mouth-speculum, consisting of upper and lower jaws, with straight bodies, spreader-bars pivotally-connecting said jaws in pairs, diagonally arranged levers, two for each side, with a locking device, and arranged to fasten to the head-halter.

FOR SALE.

A MODERATE sized practice, increasing annually. Location excellent. Splendid neighborhood, surrounded by rich farming and dairying territory. The seller about to enter other avocation. Will be sold at a very low price. Good-will, instruments, etc., etc. Address, JOURNAL OFFICE, 3452 Ludlow Street, Philadelphia, Pa.
